# Draft Genome Sequences of Four Plant Growth-Promoting Rhizobacteria Isolated from Saffron (Crocus sativus L.) Rhizosphere in Morocco

**DOI:** 10.1128/mra.01046-22

**Published:** 2023-01-12

**Authors:** Rahma Zouagui, Houda Zouagui, Mohammed Walid Chemao-Elfihri, Imane Chamkhi, Jamal Aurag, Azeddine Ibrahimi, Laila Sbabou

**Affiliations:** a Laboratory of Microbiology and Molecular Biology, Faculty of Sciences, Mohammed V University in Rabat, Rabat, Morocco; b Biotechnology Laboratory, Bioinova Research Center, Rabat Medical and Pharmacy School, Mohammed V University in Rabat, Rabat, Morocco; University of Arizona

## Abstract

We report the draft genome sequences of plant growth-promoting Rahnella perminowiae strain S11P1, *Variovorax* sp. strain S12S4, and Pseudomonas sp. strains S11A4 and S11P7, which were isolated from saffron (Crocus sativus L.) rhizosphere. Several genes were predicted to be involved in auxin production, phosphate solubilization, and other specialized functions in plant growth and defense.

## ANNOUNCEMENT

Plant growth-promoting rhizobacteria (PGPR) are free-living bacteria that colonize the rhizosphere and are capable of enhancing plant growth and defense (1). PGPR can act by suppressing disease-causing phytopathogens, modulating phytohormones levels, and increasing nutrient availability and assimilation (2). The four studied strains were isolated from saffron rhizosphere. They showed interesting plant growth-promoting traits (3) and promising performance when used as inoculants for saffron in field trials (4). These genomes are the first to be sequenced from saffron rhizosphere in Morocco and can help elucidate the beneficial plant-microbe interactions.

Root-adhering soil was sampled from Crocus sativus L. rhizosphere. The soil samples were collected from a saffron farm in the Taliouine-Taznakht region in Morocco (30°28′12.997″N, 7°46′22.479″W). The rhizospheric bacteria were isolated from 4 g of soil suspended in 16 mL of sterile sodium chloride solution (0.9%) and serially diluted. Volumes of 100 μL from each soil dilution were inoculated on Pikovskaya’s medium (5), Modi medium (6), and yeast extract-mannitol (YEM)-tryptophan medium (7) and then incubated at 28°C for 48 h. The strains were purified and stored at −80°C in Luria-Bertani (LB) medium with 40% glycerol.

The genomic DNA was extracted from an overnight liquid LB medium culture using the QIAamp genomic DNA minikit (Qiagen, Germany) and quantified with a Qubit 3.0 fluorometer. The library was prepared using the rapid barcoding sequencing kit (SQK-RBK004), and whole-genome sequencing was performed on a MinION Mk1c system (Oxford Nanopore Technologies, Oxford, UK). The four samples were multiplexed in one run and sequenced using an R9 flow cell for 72 h. The reads were base called and demultiplexed using Guppy (v. 5.0.7). The raw reads were trimmed using Porechop (v. 0.2.4) (8), *de novo* assembled with Canu (v. 2.2) (9), and polished using Racon (v. 1.5.0) (10). The annotation was carried out using the NCBI Prokaryotic Genome Annotation Pipeline (PGAP) (v. 6.2) (11) (Table 1).

**TABLE 1:** Draft genome assemblies and annotation metrics

Parameter	Finding for:			
	Rahnella perminowiae S11P1	Pseudomonas sp. strain S11P4	Pseudomonas sp. strain S11P7	*Variovorax* sp. strain S12S4
Coverage (×)	155	154	153	154
No. of reads	252,993	287,244	461,339	1,096,351
Mean read length (bp)	4,917.24	5,992.68	5,077.86	9,323.23
Read *N*_50_ (bp)^*a*^	10,903	13,917	13,338	15,886
No. of contigs	7	5	2	2
Contig *N*_50_ (bp)	4,954,455	6,315,842	6,316,889	5,907,228
GC content (%)	51.4	59.9	60.0	65.7
No. of CDSs^*b*^	5,401	5,842	5,699	5,719
No. of rRNAs	22	19	19	6
No. of tRNAs	77	76	76	57

a *N*_50_, the contig length for which 50% of the entire assembly is contained in contig no shorter than this length.

b CDSs, coding DNA sequences.

Strain identification was performed through digital DNA-DNA hybridization (dDDH) calculations using the Genome-to-Genome Distance Calculator (GGDC; https://ggdc.dsmz.de/ggdc.php) (v. 2.1, formula 2) (12) and confirmed by average nucleotide identity (ANI) analysis using pyani (v. 0.2.9) (13). Default parameters were used for all software tools.

*Rahnella* sp. strain S11P1 demonstrates an ANI value of 99.24% and a dDDH value of 93% with respect to Rahnella perminowiae type strain SL6 (GenBank accession number NZ_JAFMOS010000000). The dDDH best matches for Pseudomonas sp. strain S11P4, Pseudomonas sp. strain S11P7, and *Variovorax* sp. strain S12S4 were below the 70% threshold for species delineation (12), with similarity values of 52.2%, 52.1%, and 36.4%, respectively. Additionally, the three strains had ANI values of <95% with respect to the genomes of the type strains and thus could represent novel bacterial species (14).

The four genomes carried genes responsible for phosphate solubilization, siderophore production, and nitrogen fixation. Additionally, we predicted several genes related to auxin production, including the tryptophane synthase-encoding gene and the *ipdC* gene, which encodes indole-3-pyruvate decarboxylase, a key enzyme of the indole-3-acetic acid (IAA) pathway (15). The four PGPR strains harbor putative genes associated with biocontrol of phytopathogens, such as the genes involved in phenazine production, γ-aminobutyric acid (GABA) metabolism, and bacteriocin synthesis. Genes associated with the abiotic stress response and bacterium-plant interactions, including those for trehalose, motility, and chemotaxis, were also predicted.

### Data availability.

The data from this project are available under NCBI BioProject accession number PRJNA827450.The raw sequencing reads have been deposited in the Sequence Read Archive (SRA) under accession numbers SRX17797807 (S11P1), SRX17797806 (S11A4), SRX17797809 (S11P7), and SRX17797808 (S12S4). The genome assemblies of Rahnella perminowiae S11P1, Pseudomonas sp. strain S11A4, Pseudomonas sp. strain S11P7, and *Variovorax* sp. strain S12S4 have been deposited in GenBank under the accession numbers JALMGI020000000, JALMGH020000000, JALPKS020000000, and JALPKR020000000, respectively. The versions described in this paper are the second versions.
